# Assessment of Weight Loss and Gastrointestinal Symptoms Suggestive of Exocrine Pancreatic Dysfunction After Acute Pancreatitis

**DOI:** 10.14309/ctg.0000000000000283

**Published:** 2020-12-15

**Authors:** Anna Evans Phillips, Kohtaro Ooka, Ioannis Pothoulakis, Pedram Paragomi, Nicole Komara, Ali Lahooti, Diala Harb, Melanie Mays, Filippos Koutroumpakis, Kimberly Stello, Phil J. Greer, David C. Whitcomb, Georgios I. Papachristou

**Affiliations:** 1 Division of Gastroenterology, Hepatology, and Nutrition, Department of Medicine, University of Pittsburgh School of Medicine (UPMC), Pittsburgh, Pennsylvania, USA;; 2 Department of Medicine, MedStar Washington Hospital Center, Washington, DC, USA;; 3 Global Medical Affairs, AbbVie, Mettawa, Illinois, USA;; 4 Division of Gastroenterology, Hepatology, and Nutrition, The Ohio State University, Wexler Medical Center, Columbus, Ohio, USA.

## Abstract

Studies evaluating the natural history of exocrine pancreatic dysfunction (EPD) after acute pancreatitis (AP) are sparse. This study aims to assess incidence and predictors of weight loss and gastrointestinal (GI) symptoms suggestive of EPD 12 months after an AP episode.

**METHODS:**

Patients enrolled in the Pancreatitis-associated Risk of Organ Failure Study at the time of an AP episode were included. Weight and GI symptom data were prospectively collected by self-report at enrollment and at 3- and 12-month (windows 2–7 and 8–20) telephone follow-ups. Multivariable logistic regression was used to assess factors associated with ≥10% total body weight loss (EPD surrogate) at 12 months. A generalized estimating equation was used to measure each factor's population effect (in pounds) over 12 months after AP.

**RESULTS:**

Follow-up at 12 months in 186 patients (median age = 54 years, 46% men, 45% biliary, 65% first AP attack) revealed weight loss ≥10% from baseline, occurring in 44 patients (24%). Risk of weight loss increased with higher baseline body mass index, previous diagnosis of diabetes mellitus, and worsening AP severity (all *P* < 0.010). GI symptoms were reported in 13/31 (42%) patients at 12 months. AP severity was independently associated with ≥10% weight loss at 12 months. Over 12 months, men lost more weight than women (average 9.5 lbs); patients with severe AP lost, on average, 14 lbs.

**DISCUSSION:**

Weight loss after AP occurs in one-quarter of patients and is associated with AP severity. EPD incidence after AP is likely underappreciated. Further work is needed to assess EPD and potential for pancreatic enzyme supplementation.

## INTRODUCTION

Acute pancreatitis (AP) is among the top 3 gastrointestinal (GI) reasons for hospital admission, accounting for more than 290,000 hospitalizations and costing in excess of 2.7 billion dollars annually in the United States (1). It has been a common clinical assumption that recovery from an AP episode results in complete reconstitution of pancreatic function except in cases where large sections of the pancreas have been destroyed by necrosis (2). Therefore, most patients with AP who have an uneventful clinical course without local or systemic complications have not traditionally been followed up systematically after their discharge from inpatient care.

Recent studies have demonstrated that a subgroup of patients develop signs of exocrine pancreatic insufficiency (EPI) after resolution of their AP episode (3,4), with variable incidence rates reported from less than 24% to more than 80% (5–10). Three meta-analyses have estimated a pooled incidence of EPI between 27% and 35% during follow-up (11–13). EPI rate varied based on the diagnostic method, etiology of AP, and length of the follow-up after AP attack (7,8,14). In addition, severity of AP, occurrence of pancreatic necrosis, and history of necrosectomy (all suggestive of more extensive destruction of the gland) have been shown to correlate with higher rates of EPI compared with mild attacks (4,15–17). Recovery of pancreatic exocrine function can also occur—patients who exhibit early signs of EPI immediately after an attack have been seen to recover at varying lengths of follow-up (2).

Current guidelines for AP-related EPI evaluation do not exist. The clinical diagnosis of EPI has historically been challenging in subjects with AP, in part because there has been a lack of agreement on how to make the diagnosis definitively (18). Weight loss and GI symptoms represent the most pronounced clinical findings of exocrine pancreatic dysfunction (EPD), a term that can be used to describe a reduction of the exocrine function of the gland even in the absence of formal EPI diagnosis. In a retrospective analysis of an AP cohort, previous evaluation of GI symptoms and weight loss has been suggested as reliably reported symptoms indicative of EPD (19). Involuntary weight loss of ≥10% of normal body weight over 12 months is believed to represent protein-energy malnutrition, a condition that is often associated with a catabolic state, poor wound healing, and nutritional deficiencies (20).

We hypothesize that after an AP episode, patients may develop GI symptoms and/or clinically significant weight loss relative to baseline that are suggestive of EPD. The aims of this study were to assess patients with AP at 3 and 12 months after their episode as a surrogate for EPD to determine its prevalence and evaluate which subgroups are at higher risk of developing weight loss and symptoms suggestive of EPD.

## MATERIALS AND METHODS

### Patient population

The Pancreatitis-associated Risk of Organ Failure Study is a prospective, observational study conducted at the University of Pittsburgh Medical Center (21,22). Patients are enrolled with an AP diagnosis meeting 2 of the 3 criteria (serum lipase ≥3 times upper limit of normal, characteristic epigastric abdominal pain, or cross-sectional imaging consistent with pancreatic inflammation). Exclusion criteria include a previous diagnosis of chronic pancreatitis or suspicion of a pancreatic cancer. Enrollment for this study occurred from 2012 to 2018, with the follow-up through January 2020. Informed consent was obtained for each subject before enrollment; ethical approval was obtained by the University of Pittsburgh Institutional Review Board (PRO 08010374).

### Data collection

Demographic data, laboratory, and radiologic data were collected at enrollment. Baseline weight (in pounds) was self-reported and recorded at the time of the patient interview. Presence of pre-existing diabetes mellitus was obtained by patient interview at the time of enrollment and confirmed with the electronic medical record including evidence of previous hemoglobin A1c level ≥6.5% or use of insulin therapy or oral hypoglycemic medications as part of the home medication regimen. Active alcohol and tobacco use were defined as confirmatory response to the question(s) “Do you currently drink alcohol/smoke tobacco cigarettes?” Severity of AP was defined according to the Revised Atlanta Classification (RAC) (23).

Follow-up data including weight, new diagnosis of diabetes mellitus (DM) or formal diagnosis of EPI by a physician, and use of pancreatic enzyme replacement therapy (PERT) were collected by the patient interview at 3 months (window: 2–7 months) and 12 months (window: 8–20 months) via telephone survey and by confirmation with the electronic medical record when data were available for review. At least 5 attempts were made to contact the patient by telephone before they were declared lost to follow-up. Body mass index (BMI) was calculated by using the patient's reported height from baseline and self-reported weight at each follow-up time point.

### GI Symptom Tracker

In February 2018, a survey to elucidate symptoms of EPD called “GI Symptom Tracker” was added to the study protocol for follow-up at 3 and 12 months. The survey was developed through qualitative focus group interviews of patients with known EPI and is undergoing ongoing psychometric evaluation for establishment of validity and reliability (https://www.identifyepi.com/content/pdf/epi-gi-symptom-tracker.pdf). It consisted of the following question: “During the past 2 weeks, how often have you (a) had frequent diarrhea, (b) greasy stools, (c) loose stools, (d) felt bloated, (e) had excessive gas, (f) abdominal pain, or (g) had to rush to the bathroom in the middle of the night”? Response choices were as follows: (a) almost always, (b) often, (c) sometimes, or (d) never. Subjects who reported a positive response (>never) to any of the items qualified as having symptoms of EPD.

### Statistical analysis

Significant weight loss was defined as ≥10% of body weight compared with baseline. The primary outcome of the study was the incidence of significant weight loss at 12 months after an AP episode. Demographics, weight, BMI, etiology, active alcohol and tobacco use, recurrent attack, severity of attack, new diagnosis of DM or EPI, PERT, and GI symptoms of EPD were compared between groups with and without significant weight loss.

Categorical variables were described using frequencies and percentages. Continuous variables were presented as median and interquartile ranges (IQRs). Univariate analysis was performed to compare patients with and without significant weight loss at 3 and 12 months. A backward stepwise logistic regression model (using *P* > 0.20 as the significance level to exclude variables) was used in patients who completed the follow-up at 3 and 12 months to determine independent predictors of significant weight loss at each of the 2 intervals. The model was adjusted for age, sex, baseline BMI, etiology, active alcohol use, active cigarette smoking, recurrence of AP, severity (as measured by RAC), total hospital length of stay, and DM diagnosis before the AP attack. A two-sided Cochran-Armitage test was used to evaluate the association between weight loss and AP severity.

A generalized estimating equation (GEE) model was created using the geepack package (https://cran.r-project.org/web/packages/geepack/geepack.pdf) to evaluate associations of baseline clinical factors with longitudinal changes in weight after AP. Clinically relevant factors from the univariate analyses were included in the initial model and removed using a reverse stepwise process. The final model was selected based on the smallest quasi-likelihood under the independence model criterion values using package MuMIn (https://cran.rproject.org/web/packages/MuMIn/MuMIn.pdf) (24). The final model included sex and severity along with 2 first-order interaction terms [(sex and time) and (severity and time)]. Interaction between sex and severity were not significant. Baseline patient was a female patient with mild AP.

All statistical work was completed in R (version 3.6.2). A *P* value <0.05 was considered statistically significant.

## RESULTS

### Study cohort

**Figure 1.**
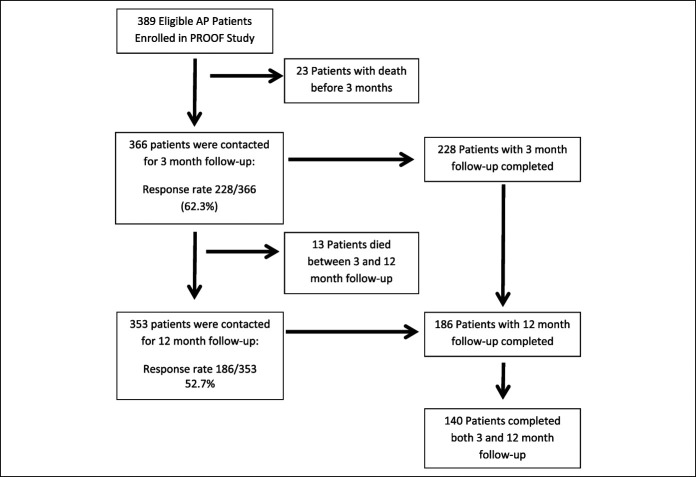
Study enrollment.

A total of 353 patients were initially contacted (Figure 1)—the final study population consisted of 186 patients (males n = 86, 46.2%) who completed the 12-month follow-up. The median age was 54 years (IQR 39–68), and median baseline BMI was 28.0 kg/m^2^ (IQR 24.7–32.9). The most common AP etiology was biliary (n = 84, 45.2%), followed by idiopathic (n = 26, 16.1%), post-endoscopic retrograde cholangiopancreatography (n = 23, 12.4%), alcohol (n = 17, 9.1%), and hypertriglyceridemia (n = 15, 8.1%). Active alcohol use was reported by 73 (39.3%) and active tobacco by 37 (19.9%) patients. An AP episode at time of enrollment was the first (index) attack of pancreatitis for 121 (65.1%) and recurrent attack for 65 (34.9%) patients. Distribution of severity of AP was mild (n = 120, 64.5%), moderate (n = 40, 21.5%), and severe (n = 26, 14.0%).

At the 12-month follow-up, ≥10% weight loss was reported by 44 patients (23.7%). A new formal diagnosis of EPI was reported by 11 (5.9%) patients, and 12 patients (6.5%) were using PERT. A new diagnosis of DM was reported by 9 (4.8%) patients. Within the subgroup of 31 patients who completed the GI Symptom Tracker, 13 (41.9%) reported GI symptoms (Table 1).

**Table 1.**
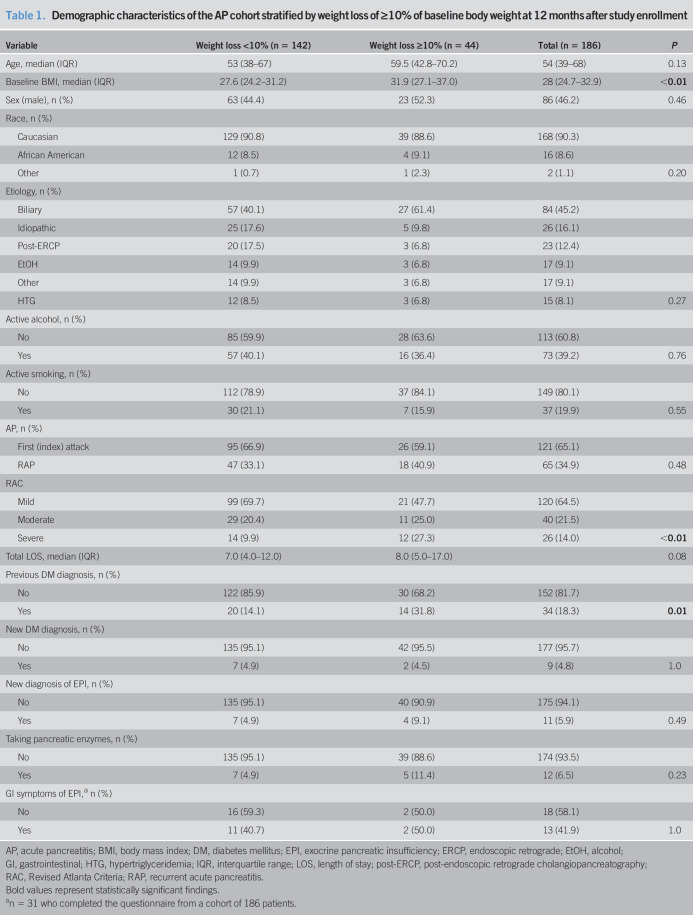
Demographic characteristics of the AP cohort stratified by weight loss of ≥10% of baseline body weight at 12 months after study enrollment

Variable	Weight loss <10% (n = 142)	Weight loss ≥10% (n = 44)	Total (n = 186)	*P*
Age, median (IQR)	53 (38–67)	59.5 (42.8–70.2)	54 (39–68)	0.13
Baseline BMI, median (IQR)	27.6 (24.2–31.2)	31.9 (27.1–37.0)	28 (24.7–32.9)	**<0.01**
Sex (male), n (%)	63 (44.4)	23 (52.3)	86 (46.2)	0.46
Race, n (%)				
Caucasian	129 (90.8)	39 (88.6)	168 (90.3)	
African American	12 (8.5)	4 (9.1)	16 (8.6)	
Other	1 (0.7)	1 (2.3)	2 (1.1)	0.20
Etiology, n (%)				
Biliary	57 (40.1)	27 (61.4)	84 (45.2)	
Idiopathic	25 (17.6)	5 (9.8)	26 (16.1)	
Post-ERCP	20 (17.5)	3 (6.8)	23 (12.4)	
EtOH	14 (9.9)	3 (6.8)	17 (9.1)	
Other	14 (9.9)	3 (6.8)	17 (9.1)	
HTG	12 (8.5)	3 (6.8)	15 (8.1)	0.27
Active alcohol, n (%)				
No	85 (59.9)	28 (63.6)	113 (60.8)	
Yes	57 (40.1)	16 (36.4)	73 (39.2)	0.76
Active smoking, n (%)				
No	112 (78.9)	37 (84.1)	149 (80.1)	
Yes	30 (21.1)	7 (15.9)	37 (19.9)	0.55
AP, n (%)				
First (index) attack	95 (66.9)	26 (59.1)	121 (65.1)	
RAP	47 (33.1)	18 (40.9)	65 (34.9)	0.48
RAC				
Mild	99 (69.7)	21 (47.7)	120 (64.5)	
Moderate	29 (20.4)	11 (25.0)	40 (21.5)	
Severe	14 (9.9)	12 (27.3)	26 (14.0)	**<0.01**
Total LOS, median (IQR)	7.0 (4.0–12.0)	8.0 (5.0–17.0)		0.08
Previous DM diagnosis, n (%)				
No	122 (85.9)	30 (68.2)	152 (81.7)	
Yes	20 (14.1)	14 (31.8)	34 (18.3)	**0.01**
New DM diagnosis, n (%)				
No	135 (95.1)	42 (95.5)	177 (95.7)	
Yes	7 (4.9)	2 (4.5)	9 (4.8)	1.0
New diagnosis of EPI, n (%)				
No	135 (95.1)	40 (90.9)	175 (94.1)	
Yes	7 (4.9)	4 (9.1)	11 (5.9)	0.49
Taking pancreatic enzymes, n (%)				
No	135 (95.1)	39 (88.6)	174 (93.5)	
Yes	7 (4.9)	5 (11.4)	12 (6.5)	0.23
GI symptoms of EPI,^a^ n (%)				
No	16 (59.3)	2 (50.0)	18 (58.1)	
Yes	11 (40.7)	2 (50.0)	13 (41.9)	1.0

AP, acute pancreatitis; BMI, body mass index; DM, diabetes mellitus; EPI, exocrine pancreatic insufficiency; ERCP, endoscopic retrograde; EtOH, alcohol; GI, gastrointestinal; HTG, hypertriglyceridemia; IQR, interquartile range; LOS, length of stay; post-ERCP, post-endoscopic retrograde cholangiopancreatography; RAC, Revised Atlanta Criteria; RAP, recurrent acute pancreatitis.

Bold values represent statistically significant findings.

a n = 31 who completed the questionnaire from a cohort of 186 patients.

### Patients with follow-ups at 3 and 12 months

A total of 140 patients (43 male, 39.8%) completed the follow-ups at 3 and 12 months (baseline characteristics, Table 2). Of the 38 patients (27.1%) who showed ≥10% weight loss at the 3-month follow-up, 16 (42.1%) regained weight by the 12-month follow-up and no longer met the 10% threshold. By contrast, 10 subjects who had not met the 10% threshold at 3 months lost more weight to reach the 10% threshold at the 12-month follow-up.

**Table 2.**
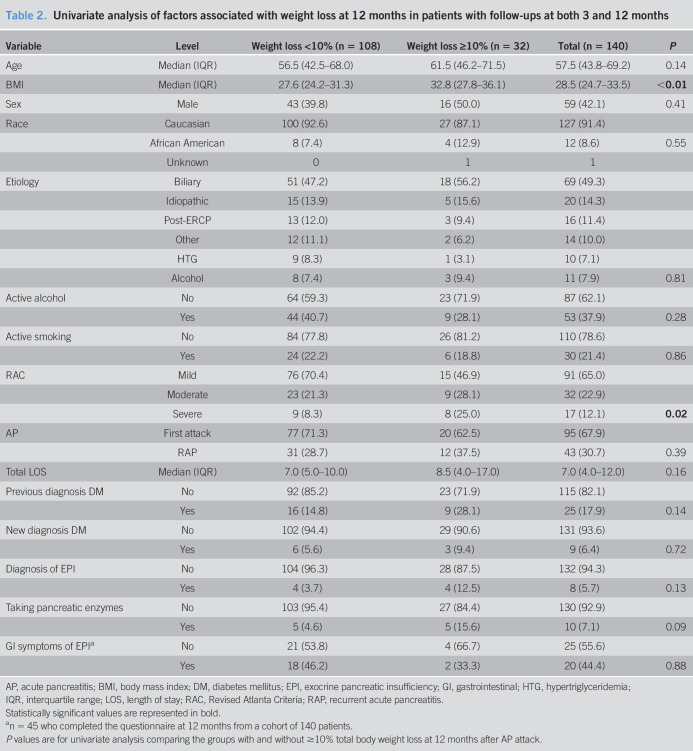
Univariate analysis of factors associated with weight loss at 12 months in patients with follow-ups at both 3 and 12 months

Variable	Level	Weight loss <10% (n = 108)	Weight loss ≥10% (n = 32)	Total (n = 140)	*P*
Age	Median (IQR)	56.5 (42.5–68.0)	61.5 (46.2–71.5)	57.5 (43.8–69.2)	0.14
BMI	Median (IQR)	27.6 (24.2–31.3)	32.8 (27.8–36.1)	28.5 (24.7–33.5)	**<0.01**
Sex	Male	43 (39.8)	16 (50.0)	59 (42.1)	0.41
Race	Caucasian	100 (92.6)	27 (87.1)	127 (91.4)	
	African American	8 (7.4)	4 (12.9)	12 (8.6)	0.55
	Unknown	0	1	1	
Etiology	Biliary	51 (47.2)	18 (56.2)	69 (49.3)	
	Idiopathic	15 (13.9)	5 (15.6)	20 (14.3)	
	Post-ERCP	13 (12.0)	3 (9.4)	16 (11.4)	
	Other	12 (11.1)	2 (6.2)	14 (10.0)	
	HTG	9 (8.3)	1 (3.1)	10 (7.1)	
	Alcohol	8 (7.4)	3 (9.4)	11 (7.9)	0.81
Active alcohol	No	64 (59.3)	23 (71.9)	87 (62.1)	
	Yes	44 (40.7)	9 (28.1)	53 (37.9)	0.28
Active smoking	No	84 (77.8)	26 (81.2)	110 (78.6)	
	Yes	24 (22.2)	6 (18.8)	30 (21.4)	0.86
RAC	Mild	76 (70.4)	15 (46.9)	91 (65.0)	
	Moderate	23 (21.3)	9 (28.1)	32 (22.9)	
	Severe	9 (8.3)	8 (25.0)	17 (12.1)	**0.02**
AP	First attack	77 (71.3)	20 (62.5)	95 (67.9)	
	RAP	31 (28.7)	12 (37.5)	43 (30.7)	0.39
Total LOS	Median (IQR)	7.0 (5.0–10.0)	8.5 (4.0–17.0)	7.0 (4.0–12.0)	0.16
Previous diagnosis DM	No	92 (85.2)	23 (71.9)	115 (82.1)	
	Yes	16 (14.8)	9 (28.1)	25 (17.9)	0.14
New diagnosis DM	No	102 (94.4)	29 (90.6)	131 (93.6)	
	Yes	6 (5.6)	3 (9.4)	9 (6.4)	0.72
Diagnosis of EPI	No	104 (96.3)	28 (87.5)	132 (94.3)	
	Yes	4 (3.7)	4 (12.5)	8 (5.7)	0.13
Taking pancreatic enzymes	No	103 (95.4)	27 (84.4)	130 (92.9)	
	Yes	5 (4.6)	5 (15.6)	10 (7.1)	0.09
GI symptoms of EPI^a^	No	21 (53.8)	4 (66.7)	25 (55.6)	
	Yes	18 (46.2)	2 (33.3)	20 (44.4)	0.88

AP, acute pancreatitis; BMI, body mass index; DM, diabetes mellitus; EPI, exocrine pancreatic insufficiency; GI, gastrointestinal; HTG, hypertriglyceridemia; IQR, interquartile range; LOS, length of stay; RAC, Revised Atlanta Criteria; RAP, recurrent acute pancreatitis.

Statistically significant values are represented in bold.

a n = 45 who completed the questionnaire at 12 months from a cohort of 140 patients.

*P* values are for univariate analysis comparing the groups with and without ≥10% total body weight loss at 12 months after AP attack.

### Univariate analysis of the 12-month follow-up

Compared with the 142 patients (44.4% male) without significant weight loss, the 44 patients (52.3% men) with ≥10% weight loss at 12 months were more likely to have a previous diagnosis of DM (31.8 vs 14.1, *P* = 0.01) or have severe disease per RAC (severe AP in 27.3% vs 9.9%, *P* < 0.01). No significant differences in the rates of weight loss were associated with age, AP etiology, active alcohol or active tobacco use, first attack vs recurrence, new diagnosis of EPI, or PERT use. In the subgroup analysis of patients who filled out the GI Symptom Tracker, no significant difference was seen in the proportion of patients experiencing GI symptoms of EPD (Table 1).

### Multivariable analysis

Multivariable logistic regression analysis controlling for age, BMI, sex, previous diagnosis of DM, etiology, active alcohol and smoking, first vs recurrent AP, AP severity, and total length of stay showed AP severity as the only significant independent predictor of ≥10% weight loss at the 12-month follow-up (Table 3).

**Table 3.**
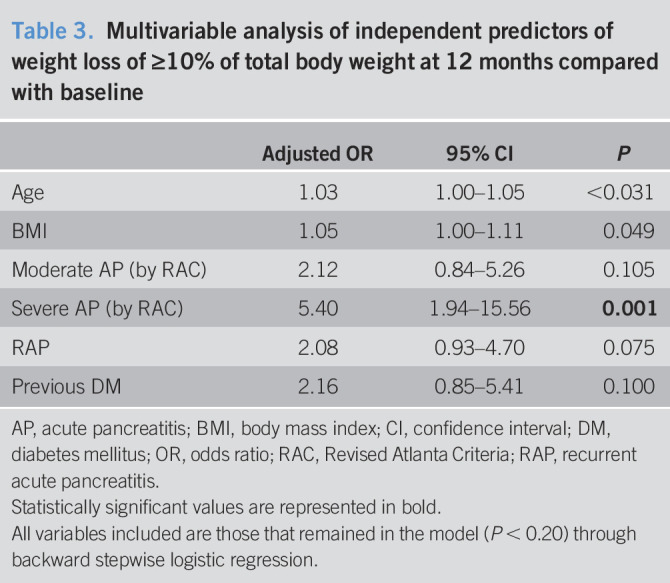
Multivariable analysis of independent predictors of weight loss of ≥10% of total body weight at 12 months compared with baseline

	Adjusted OR	95% CI	*P*
Age	1.03	1.00–1.05	<0.031
BMI	1.05	1.00–1.11	0.049
Moderate AP (by RAC)	2.12	0.84–5.26	0.105
Severe AP (by RAC)	5.40	1.94–15.56	**0.001**
RAP	2.08	0.93–4.70	0.075
Previous DM	2.16	0.85–5.41	0.100

AP, acute pancreatitis; BMI, body mass index; CI, confidence interval; DM, diabetes mellitus; OR, odds ratio; RAC, Revised Atlanta Criteria; RAP, recurrent acute pancreatitis.

Statistically significant values are represented in bold.

All variables included are those that remained in the model (*P* < 0.20) through backward stepwise logistic regression.

### Modeling of weight changes after AP

In a GEE model with baseline patient represented by a female patient with mild AP, the model revealed that men lost on average 9.5 lbs more than women at the 12-month follow-up (*P* < 0.015). In the same model, patients with moderate AP lost on average 9.5 lbs at 3 months but recovered to a loss of 4.4 lbs at the 12-month follow-up. Patients with severe AP, by comparison, lost on average 24.2 lbs at 3 months and 14.19 lbs at 12 months after the AP episodes (all *P* < 0.05, Table 4).

**Table 4.**
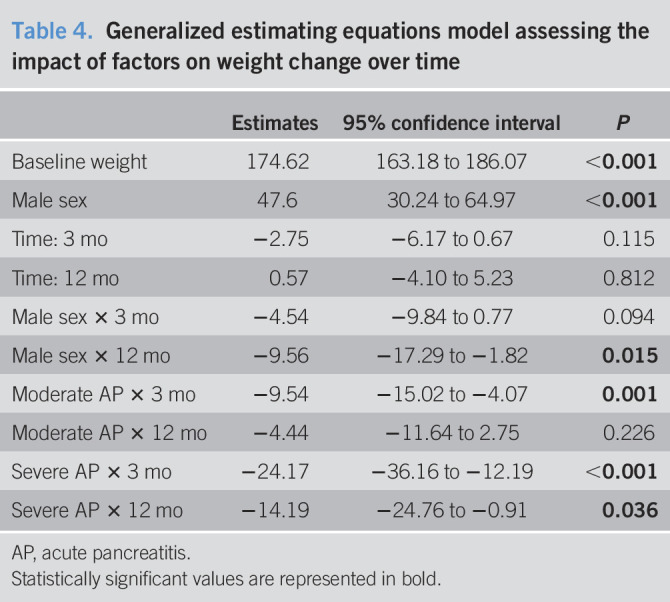
Generalized estimating equations model assessing the impact of factors on weight change over time

	Estimates	95% confidence interval	*P*
Baseline weight	174.62	163.18 to 186.07	**<0.001**
Male sex	47.6	30.24 to 64.97	**<0.001**
Time: 3 mo	−2.75	−6.17 to 0.67	0.115
Time: 12 mo	0.57	−4.10 to 5.23	0.812
Male sex × 3 mo	−4.54	−9.84 to 0.77	0.094
Male sex × 12 mo	−9.56	−17.29 to −1.82	**0.015**
Moderate AP × 3 mo	−9.54	−15.02 to −4.07	**0.001**
Moderate AP × 12 mo	−4.44	−11.64 to 2.75	0.226
Severe AP × 3 mo	−24.17	−36.16 to −12.19	**<0.001**
Severe AP × 12 mo	−14.19	−24.76 to −0.91	**0.036**

AP, acute pancreatitis.

Statistically significant values are represented in bold.

### Patients with the follow-up at 3 months

In the cohort of patients who followed up at 3 months (n = 228 total, 98 [43%] men; baseline characteristics in Supplementary Table 1, see Supplementary Digital Content 1, http://links.lww.com/CTG/A468), 80 patients (35%) reported ≥10% weight loss from baseline. New DM was reported by 15 patients (6.6%), and new diagnosis of EPI was reported by 17 patients (7.5%), with a total of 19 patients (8.3) using PERT. Higher BMI and previous DM diagnosis were independently associated with weight loss at 3 months, but not at 12 months. Within the 46 patients who completed the GI Symptom Tracker, 7 patients (15.2%) reported GI symptoms of EPD. Univariate analysis and multivariable logistic regression revealed overall similar results (see Supplementary Tables 1 to 3, Supplementary Digital Contents 1 to 3, http://links.lww.com/CTG/A468, http://links.lww.com/CTG/A469, and 3 http://links.lww.com/CTG/A470) to the primary outcomes of the study measured at the 12-month follow up.

## DISCUSSION

In this prospective analysis of a large cohort of patients with AP, we demonstrated that 24% experienced significant weight loss at 12 months after an AP episode. Of those who lost weight during the first 3 months after an AP episode, >40% returned to baseline at 12 months and approximately 25% who had ≥10% weight loss at 12 months had not experienced this by 3 months. The GEE model further revealed that men lost on average 9.5 lbs more than women at the 12-month follow-up. Although subsets of patients may experience separate trajectories of EPD symptoms, severity of AP was the only independent predictor of ≥10% weight loss at the 12-month follow-up.

Weight loss and GI symptoms remain the most frequent clinical manifestations of EPD and represent a good screening mechanism for providers concerned about its occurrence in the months after AP attack. The lack of agreement on a definitive method for diagnosis of EPI in subjects with AP, and the cumbersome and changing nature of available diagnostic testing for EPI have created major barriers to accurate and timely diagnosis (18,19,25). The gold standard for EPI diagnosis—duodenal aspiration after IV secretin administration—is expensive, logistically challenging, and largely unavailable in clinical practice (26). Indirect testing such as the 72-hour fecal fat collection is highly sensitive but not specific for EPI and is cumbersome for both the patient and the researcher and therefore uncommonly used. Fecal elastase-1 (FE-1) has become widely used in outpatient clinics; however, despite high sensitivity and specificity in moderate and severe EPI, it remains unreliable in mild EPI (27–31). Furthermore, FE-1 testing should be obtained while the patient is eating a normal diet. In the acute inpatient setting, it is unlikely that patients who have just experienced an attack of AP are back to eating their normal diet. For these reasons FE-1, although widely used, is a test with significant limitations.

In AP, the natural history of EPI remains ill defined. In chronic pancreatitis (CP), EPI is a well-described phenomenon, but its prevalence varies widely, with estimates ranging from 21 to 94% of patients and increasing with disease duration (32–35). Expert opinion has suggested that EPI manifestation in CP occurs after approximately 90% of endogenous pancreatic enzyme secretion is lost (36). EPI symptoms have been shown to improve in CP clinical trials with PERT (37–39). EPI after pancreatic surgery has also been described and perprotocol analysis of a recent postpancreaticoduodenectomy cohort of 164 patients showed a significant difference in weight at 3 months postoperatively with weight gain in those treated with PERT and weight loss in those treated with placebo (40).

The findings of our study of EPD after AP are consistent with the previous estimates of EPI burden after AP and confirm the idea that subgroups of patients experience weight loss suggestive of EPD at different points after their attack (11–13,41). One group of patients experienced early weight loss at 3 months and then recovered by 12 months, whereas another maintained their weight through 3 months then lost weight by 12 months. Higher BMI and previous DM diagnosis were independently associated with weight loss at 3 months, but not at 12 months, suggesting that something about these characteristics (more rapid loss of excess body fat or poorer control of diabetes in the weeks after AP attack) may result in rapid weight loss with eventual recovery. Certain subsets of patients may benefit from PERT therapy or more individualized nutritional plans at different points after their attack of AP.

The low index of suspicion for EPD after AP may lead to misdiagnosis of true EPI, underdiagnosis, delay of treatment, and adverse consequences of the patients' nutritional health. Patients with AP and subsequent EPI have been found to develop micronutrient deficiencies, e.g., vitamin D, iron, folic acid, and vitamin B12 (10). It remains unclear after AP when exactly EPI requires PERT intervention—during the acute phase, immediately after resolution of AP, or long after its resolution. There is a dearth of clinical trial evidence showing PERT benefit in this population nor any guidelines on optimal frequency or dose of PERT in patients with AP. To date, there is only one small double-blinded clinical trial in approximately 50 patients of PERT during the refeeding period of AP (42). This study showed improved quality of life compared with placebo but was limited by the small number of patients and short study duration. The study also showed a less severe weight loss in PERT-treated patients, but this difference did not reach statistical significance. In a separate observational study using a survey completed 24–36 months after an episode of severe AP, 76% of patients reported having unintentional weight loss and ongoing GI symptoms, including abdominal pain and frequent diarrhea, and 20% of patients required regular PERT (9). This study was limited by the inclusion of only patients with severe AP, which represent a minority of all subjects with AP. In our study, the incidence of EPI diagnoses and PERT therapy were assessed by the patient report, but whether the diagnosis was made and therapy started because of weight loss or to prevent weight loss was unknown. Additional trials are needed to determine the optimal dose and timing of PERT therapy after an attack of AP.

Weight loss after AP may also be related to the development of pancreatic cancer. Approximately 10% of new pancreatic cancer diagnoses may first manifest with an episode of AP (43). An increased risk of pancreatic cancer has been associated with a previous diagnosis of AP after 5 years of follow-up in a large cohort study based on administrative data (44). Chronic pancreatitis is a known independent risk factor for pancreatic cancer (45,46). In patients who develop diabetes, the risk of pancreatic cancer is even higher when combined with either form of pancreatitis (47). In the cohort of patients initially invited to participate in our study, 5 patients developed pancreatic cancer and one developed distal cholangiocarcinoma during the follow-up. Of the patients who developed cancer, 2 who developed pancreatic cancer completed the 3-month follow-up (before cancer diagnosis)—one had ≥10% weight loss and one did not. All subjects who developed cancer were lost to follow up by the 12-month time point. Microbiome alterations after AP have also been proposed to influence EPD, although additional work on this link needs to be performed to understand mechanisms of microbiota influence on abdominal symptoms and weight loss after AP (48).

There are several limitations of this study. The follow-up response rate at 3 months was 228/366 (62.3%), 12 months was 186/353 (52.7%), and both time points was 140/353 (39.7%). Completion of the follow-up for all patients at both time points was optimized by the wide follow-up windows, but we acknowledge the presence of potential bias toward patients motivated to participate because of continued symptoms. Barriers to follow-up included patient interval death (related or unrelated to AP), inability to participate in phone survey (placement in nursing homes without personal telephone or inability to hear questions over phone), and unwillingness to participate. It is further noted that patients with AP with alcohol etiology followed up at lower rates—they were originally 15.8% of the eligible cohort for follow-up, but only made up 9.1% of the 12-month cohort. Weight data were obtained by the patient report throughout the study for consistency. Although the collection directly from patients has the potential to introduce error, it also allows for timely reporting from patients in their natural environment, which is rare and important data to obtain for patients who have experienced mild or uncomplicated AP. Given that weight loss is an easy-to-establish sign of malnutrition and in this study cohort is likely due to EPD, a threshold of ≥10% from baseline was chosen to allow for less severe weight loss, which might be impacted by the factors of residual abdominal pain and loss of appetite. Given the high level of our chosen weight loss threshold, patients with less severe weight loss due to EPD may not be detected; we suspect that the actual degree of EPD in our patient population may be higher than estimated. A formal diagnosis of CP was not able to be evaluated in patients who exhibited EPD symptoms because of the limitations of telephone follow-ups. The GI Symptom Tracker was introduced at a later phase in the study; thus, it was only applied in a subgroup of the cohort. Finally, we did not examine the use of pancreatic enzymes as a predictor of weight loss. Interpretation of an independent association with weight loss was not possible because PERT may be prescribed by providers for either prevention or therapy.

In a longitudinal cohort of patients followed after an AP attack, approximately 24% experienced weight loss of ≥10% from baseline at the 12-month follow-up time point to which EPD was a likely contributor. Increasing AP severity was independently associated with greater risk of weight loss at 12 months. A GEE model showed that men were likely to lose 9.5 lbs more than women at 12 months after AP attack, that patients with moderate AP lost a moderate amount of weight (9.5 lbs at 3 and 4.4 lbs at 12 months), and that patient with severe AP lost the most significant weight (24.2 lbs at 3 months and 14.2 lbs at 12 months). Large prospective trials collecting stool and nutritional data at predefined time intervals post-AP are needed to assess the natural history and prevalence of EPD, as well as potential for PERT intervention.

## CONFLICTS OF INTEREST

**Guarantor of the article:** Georgios I. Papachristou, MD, PhD.

**Specific author contributions:** A.E.P.: drafting of the manuscript, study design, analysis and interpretation of the data, and critical revision of the manuscript. K.O.: acquisition of data, analysis and interpretation of the data, and revision of the manuscript. I.P.: acquisition of data and critical review of the manuscript. P.P.: study design, acquisition of data, revision of the manuscript, and study design. N.K.: acquisition of data and drafting and critical revision of the manuscript. A.L.: critical revision of the manuscript. D.H.: critical revision of the manuscript. M.M.: acquisition of data and revising the manuscript. F.K.: acquisition of data and critical revision of the manuscript. K.S.: acquisition of data and revising the manuscript. P.J.G.: statistical analysis and interpretation of the data. D.C.W.: critical revision of the manuscript. G.I.P.: study design, analysis and interpretation of the data, and drafting and critical revision of the manuscript for important intellectual content. All authors approved of the final version of the manuscript.

**Financial support:** All elements of the study were performed at the University of Pittsburgh. The study was supported by an Investigator Initiated grant funded by AbbVie.

**Potential competing of interests:** None to report.

**Study Highlights**

**WHAT IS KNOWN**

✓ Patients can experience gastrointestinal (GI) symptoms and digestive symptoms after an episode of acute pancreatitis (AP).
✓ The incidence and risk factors for significant weight loss and GI symptoms after AP is unknown.

**WHAT IS NEW HERE**

✓ Significant weight loss of ≥10% of baseline weight occurs in approximately 24% of patients after AP.
✓ GI symptoms suggestive of exocrine pancreatic dysfunction (EPD) occur in approximately 42% of patients after AP.
✓ Severity of AP is an independent predictor of significant weight loss after AP.

**TRANSLATIONAL IMPACT**

✓ Detection of post-AP EPD may be enhanced by simple screening with weight loss and assessment of new GI symptoms before confirmation with diagnostic testing.
✓ Increased rate of diagnosis of post-AP EPD may improve the patients' health in the year after AP.

## Supplementary Material

SUPPLEMENTARY MATERIAL

## References

[1] Peery AF Crockett SD Murphy CC Burden and cost of gastrointestinal, liver, and pancreatic diseases in the United States: Update 2018. Gastroenterology 2019;156(1):254–72.e1. 30315778 10.1053/j.gastro.2018.08.063 PMC6689327

[2] Uomo G Gallucci F Madrid E Pancreatic functional impairment following acute necrotizing pancreatitis: Long-term outcome of a non-surgically treated series. Dig Liver Dis 2010;42(2):149–52. 19836318 10.1016/j.dld.2009.08.007

[3] Seidensticker F Otto J Lankisch PG Recovery of the pancreas after acute pancreatitis is not necessarily complete. Int J Pancreatol 1995;17(3):225–9. 7642969 10.1007/BF02785818

[4] Symersky T van Hoorn B Masclee AA The outcome of a long-term follow-up of pancreatic function after recovery from acute pancreatitis. JOP 2006;7(5):447–53. 16998241

[5] Andersson B Pendse ML Andersson R Pancreatic function, quality of life and costs at long-term follow-up after acute pancreatitis. World J Gastroenterol 2010;16(39):4944–51. 20954281 10.3748/wjg.v16.i39.4944 PMC2957603

[6] Migliori M Pezzilli R Tomassetti P Exocrine pancreatic function after alcoholic or biliary acute pancreatitis. Pancreas 2004;28(4):359–63. 15097850 10.1097/00006676-200405000-00001

[7] Bozkurt T Maroske D Adler G Exocrine pancreatic function after recovery from necrotizing pancreatitis. Hepatogastroenterology 1995;42(1):55–8. 7782037

[8] Gupta R Wig JD Bhasin DK Severe acute pancreatitis: The life after. J Gastrointest Surg 2009;13(7):1328–36. 19415400 10.1007/s11605-009-0901-z

[9] Hochman D Louie B Bailey R Determination of patient quality of life following severe acute pancreatitis. Can J Surg 2006;49(2):101–6. 16630420 PMC3207533

[10] Vujasinovic M Tepes B Makuc J Pancreatic exocrine insufficiency, diabetes mellitus and serum nutritional markers after acute pancreatitis. World J Gastroenterol 2014;20(48):18432–8. 25561813 10.3748/wjg.v20.i48.18432 PMC4277983

[11] Hollemans RA Hallensleben NDL Mager DJ Pancreatic exocrine insufficiency following acute pancreatitis: Systematic review and study level meta-analysis. Pancreatology 2018;18(3):253–62. 29482892 10.1016/j.pan.2018.02.009

[12] Huang W de la Iglesia-Garcia D Baston-Rey I Exocrine pancreatic insufficiency following acute pancreatitis: Systematic review and meta-analysis. Dig Dis Sci 2019;64(7):1985–2005. 31161524 10.1007/s10620-019-05568-9 PMC6584228

[13] Das SL Kennedy JI Murphy R Relationship between the exocrine and endocrine pancreas after acute pancreatitis. World J Gastroenterol 2014;20(45):17196–205. 25493036 10.3748/wjg.v20.i45.17196 PMC4258592

[14] Pezzilli R Simoni P Casadei R Exocrine pancreatic function during the early recovery phase of acute pancreatitis. Hepatobiliary Pancreat Dis Int 2009;8(3):316–9. 19502175

[15] Bavare C Prabhu R Supe A Early morphological and functional changes in pancreas following necrosectomy for acute severe necrotizing pancreatitis. Indian J Gastroenterol 2004;23(6):203–5. 15627657

[16] Boreham B Ammori BJ A prospective evaluation of pancreatic exocrine function in patients with acute pancreatitis: Correlation with extent of necrosis and pancreatic endocrine insufficiency. Pancreatology 2003;3(4):303–8. 12890992 10.1159/000071768

[17] Tsiotos GG Luque-de Leon E Sarr MG Long-term outcome of necrotizing pancreatitis treated by necrosectomy. Br J Surg 1998;85(12):1650–3. 9876068 10.1046/j.1365-2168.1998.00950.x

[18] Abu-El-Haija M Conwell DL Pancreatic insufficiency: What is the gold standard? Gastrointest Endosc Clin N Am 2018;28(4):521–8. 30241641 10.1016/j.giec.2018.05.004

[19] Durie P Baillargeon JD Bouchard S Diagnosis and management of pancreatic exocrine insufficiency (PEI) in primary care: Consensus guidance of a Canadian expert panel. Curr Med Res Opin 2018;34(1):25–33. 28985688 10.1080/03007995.2017.1389704

[20] Collins N Protein-energy malnutrition and involuntary weight loss: Nutritional and pharmacological strategies to enhance wound healing. Expert Opin Pharmacother 2003;4(7):1121–40. 12831338 10.1517/14656566.4.7.1121

[21] Koutroumpakis E Slivka A Furlan A Management and outcomes of acute pancreatitis patients over the last decade: A US tertiary-center experience. Pancreatology 2017;17(1):32–40. 28341116 10.1016/j.pan.2016.10.011

[22] Machicado JD Gougol A Stello K Acute pancreatitis has a long-term deleterious effect on physical health related quality of life. Clin Gastroenterol Hepatol 2017;15(9):1435–43.e2. 28579182 10.1016/j.cgh.2017.05.037

[23] Banks PA Bollen TL Dervenis C Classification of acute pancreatitis—2012: Revision of the Atlanta classification and definitions by international consensus. Gut 2013;62(1):102–11. 23100216 10.1136/gutjnl-2012-302779

[24] Pan W Akaike's information criterion in generalized estimating equations. Biometrics 2001;57(1):120–5. 11252586 10.1111/j.0006-341x.2001.00120.x

[25] Pezzilli R Andriulli A Bassi C Exocrine pancreatic insufficiency in adults: A shared position statement of the Italian Association for the Study of the Pancreas. World J Gastroenterol 2013;19(44):7930–46. 24307787 10.3748/wjg.v19.i44.7930 PMC3848141

[26] Leeds JS Oppong K Sanders DS The role of fecal elastase-1 in detecting exocrine pancreatic disease. Nat Rev Gastroenterol Hepatol 2011;8(7):405–15. 21629239 10.1038/nrgastro.2011.91

[27] Loser C Mollgaard A Folsch UR Faecal elastase 1: A novel, highly sensitive, and specific tubeless pancreatic function test. Gut 1996;39(4):580–6. 8944569 10.1136/gut.39.4.580 PMC1383273

[28] Stein J Jung M Sziegoleit A Immunoreactive elastase I: Clinical evaluation of a new noninvasive test of pancreatic function. Clin Chem 1996;42(2):222–6. 8595714

[29] Lankisch PG Schmidt I Konig H Faecal elastase 1: Not helpful in diagnosing chronic pancreatitis associated with mild to moderate exocrine pancreatic insufficiency. Gut 1998;42(4):551–4. 9616319 10.1136/gut.42.4.551 PMC1727065

[30] Glasbrenner B Schon A Klatt S Clinical evaluation of the faecal elastase test in the diagnosis and staging of chronic pancreatitis. Eur J Gastroenterol Hepatol 1996;8(11):1117–20. 8944376 10.1097/00042737-199611000-00016

[31] Hardt PD Marzeion AM Schnell-Kretschmer H Fecal elastase 1 measurement compared with endoscopic retrograde cholangiopancreatography for the diagnosis of chronic pancreatitis. Pancreas 2002;25(1):e6–9. 12131782 10.1097/00006676-200207000-00004

[32] Dumasy V Delhaye M Cotton F Fat malabsorption screening in chronic pancreatitis. Am J Gastroenterol 2004;99(7):1350–4. 15233677 10.1111/j.1572-0241.2004.30661.x

[33] Li BR Pan J Du TT Risk factors for steatorrhea in chronic pancreatitis: A cohort of 2,153 patients. Sci Rep 2016;6:21381. 26877248 10.1038/srep21381 PMC4753434

[34] Ammann RW Buehler H Muench R Differences in the natural history of idiopathic (nonalcoholic) and alcoholic chronic pancreatitis. A comparative long-term study of 287 patients. Pancreas 1987;2(4):368–77. 3628234 10.1097/00006676-198707000-00002

[35] Levy P Dominguez-Munoz E Imrie C Epidemiology of chronic pancreatitis: Burden of the disease and consequences. United European Gastroenterol J 2014;2(5):345–54. 10.1177/2050640614548208 PMC4212500 25360312

[36] Forsmark CE Management of chronic pancreatitis. Gastroenterology 2013;144(6):1282–91.e3. 23622138 10.1053/j.gastro.2013.02.008

[37] D'Haese JG Ceyhan GO Demir IE Pancreatic enzyme replacement therapy in patients with exocrine pancreatic insufficiency due to chronic pancreatitis: A 1-year disease management study on symptom control and quality of life. Pancreas 2014;43(6):834–41. 24717829 10.1097/MPA.0000000000000131

[38] Ramesh H Reddy N Bhatia S A 51-week, open-label clinical trial in India to assess the efficacy and safety of pancreatin 40000 enteric-coated minimicrospheres in patients with pancreatic exocrine insufficiency due to chronic pancreatitis. Pancreatology 2013;13(2):133–9. 23561971 10.1016/j.pan.2013.01.009

[39] Gubergrits N Malecka-Panas E Lehman GA A 6-month, open-label clinical trial of pancrelipase delayed-release capsules (Creon) in patients with exocrine pancreatic insufficiency due to chronic pancreatitis or pancreatic surgery. Aliment Pharmacol Ther 2011;33(10):1152–61. 21418260 10.1111/j.1365-2036.2011.04631.x

[40] Kim H Yoon YS Han Y Effects of pancreatic enzyme replacement therapy on body weight and nutritional assessments after pancreatoduodenectomy in a randomized trial. Clin Gastroenterol Hepatol 2020;18(4):926–34.e4. 31520730 10.1016/j.cgh.2019.08.061

[41] Sand J Nordback I Acute pancreatitis: Risk of recurrence and late consequences of the disease. Nat Rev Gastroenterol Hepatol 2009;6(8):470–7. 19581905 10.1038/nrgastro.2009.106

[42] Kahl S Schutte K Glasbrenner B The effect of oral pancreatic enzyme supplementation on the course and outcome of acute pancreatitis: A randomized, double-blind parallel-group study. JOP 2014;15(2):165–74. 24618443 10.6092/1590-8577/797

[43] Munigala S Kanwal F Xian H Increased risk of pancreatic adenocarcinoma after acute pancreatitis. Clin Gastroenterol Hepatol 2014;12(7):1143–50.e1. 24440214 10.1016/j.cgh.2013.12.033

[44] Kirkegard J Cronin-Fenton D Heide-Jorgensen U Acute pancreatitis and pancreatic cancer risk: A nationwide matched-cohort study in Denmark. Gastroenterology 2018;154(6):1729–36. 29432727 10.1053/j.gastro.2018.02.011

[45] Lowenfels AB Maisonneuve P Cavallini G Pancreatitis and the risk of pancreatic cancer. International Pancreatitis Study Group. N Engl J Med 1993;328(20):1433–7. 8479461 10.1056/NEJM199305203282001

[46] Maisonneuve P Lowenfels AB Bueno-de-Mesquita HB Past medical history and pancreatic cancer risk: Results from a multicenter case-control study. Ann Epidemiol 2010;20(2):92–8. 20123159 10.1016/j.annepidem.2009.11.010

[47] Cho J Scragg R Petrov MS Postpancreatitis diabetes confers higher risk for pancreatic cancer than type 2 diabetes: Results from a nationwide cancer registry. Diabetes Care 2020;43(9):2106–12. 32616613 10.2337/dc20-0207

[48] Petrov MS Metabolic trifecta after pancreatitis: Exocrine pancreatic dysfunction, altered gut microbiota, and new-onset diabetes. Clin Transl Gastroenterol 2019;10(10):e00086. 31609744 10.14309/ctg.0000000000000086 PMC6884355

