# Supplementary material for: Assessment of Weight Loss and Gastrointestinal Symptoms Suggestive of Exocrine Pancreatic Dysfunction After Acute Pancreatitis
Source: Clin Transl Gastroenterol. 2020 Dec 15;11(12):e00283. doi: 10.14309/ctg.0000000000000283 (PMC7743841; doi:10.14309/ctg.0000000000000283)
Supplement: SUPPLEMENTARY MATERIAL [file ct9-11-e00283-s001.docx]

**Supplementary Table 1:** Demographic characteristics of cohort stratified by weight loss of ≥ 10% of baseline at 3 months after study enrollment

| **Variable** | **No Wt loss**  **(n=148)** | **Wt Loss ≥ 10%**  **(n=80)** | **Total (n=228)** | **p-value** |
| --- | --- | --- | --- | --- |
| Age (Median, IQR) | 51.5 (38, 68) | 58 (43.8, 68.2) | 55 (39, 68) | 0.15 |
| BMI (Median, IQR) | 27.8 (24.1, 32.6) | 31.9 (25.8, 36.7) | 28.7 (24.7, 34.8) | **<0.01** |
| Sex (n=male, %) | 67 (45.3) | 31 (38.8) | 98 (43.0) | 0.42 |
| Race |  |  |  |  |
| Caucasian | 129 (87.2) | 72 (90.0) | 201 (88.2) |  |
| African American | 16 (10.8) | 7 (8.8) | 23 (10.1) |  |
| Other | 3 (2.0) | 1 (1.2) | 4 (1.8) | 0.87 |
| Etiology Biliary | 64 (43.2) | 33 (41.2) | 97 (42.5) |  |
| EtOH | 24 (16.2) | 11 (13.8) | 35 (15.4) |  |
| Post-ERCP | 20 (13.5) | 8 (10.0) | 28 (12.3) |  |
| Idiopathic | 17 (11.5) | 14 (17.5) | 31 (13.6) |  |
| Other | 13 (8.8) | 6 (7.5) | 19 (8.3) |  |
| Hypertriglyceridemia | 10 (6.8) | 8 (10.0) | 18 (7.9) | 0.71 |
| Active Drinker No | 80 (54.1) | 50 (62.5) | 130 (57.0) |  |
| Yes | 68 (45.9) | 30 (37.5) | 98 (43.0) | 0.28 |
| Active Smoker No | 108 (73.0) | 60 (75.0) | 168 (73.7) |  |
| Yes | 40 (27.0) | 20 (25.0) | 60 (26.3) | 0.90 |
| AP Index Attack | 92 (62.2) | 48 (0.60) | 140 (61.4) |  |
| RAP | 56 (37.8) | 32 (0.40) | 88 (38.6) | 0.87 |
| RAC Mild | 100 (67.6) | 46 (57.5) | 146 (64.0) |  |
| Moderate | 38 (25.7) | 21 (26.3) | 59 (25.9) |  |
| Severe | 10 (6.7) | 13 (16.2) | 23 (10.1) | 0.06 |
| Total LOS (Median, IQR) | 6.0 (4.0, 9.0) | 7.5 (5.0, 15.0) | 7.0 (4.0, 12.0) | **<0.01** |
| Prior Diagnosis of DM No | 129 (87.2) | 59 (73.8) | 188 (82.5) |  |
| Yes | 19 (12.8) | 21 (26.2) | 40 (17.5) | **0.02** |
| New Diagnosis of DM No | 143 (96.6) | 70 (87.5) | 213 (93.4) |  |
| Yes | 5 (3.4) | 10 (12.5) | 15 (6.6) | **0.02** |
| Diagnosis of EPI No | 143 (96.6) | 68 (85.0) | 211 (92.5) |  |
| Yes | 5 (3.4) | 12 (15.0) | 17 (7.5) | **<0.01** |
| Taking Panc Enzymes No | 143 (96.6) | 66 (82.5) | 209(91.7) |  |
| Yes | 5 (3.4) | 14 (17.5) | 19 (8.3) | **<0.01** |
| GI.Symptoms of EPI* No | 22 (91.7) | 17 (77.3) | 39 (84.8) |  |
| Yes | 2 (8.3) | 5 (22.7) | 7 (15.2) | 0.34 |

* *n=46 who completed questionnaire from cohort of 228. BMI: Body Mass Index; AP: Acute Pancreatitis;RAP: Recurrent Acute Pancreatitis; RAC: Revised Atlanta Criteria; LOS: Length of Stay; DM: Diabetes Mellitus; EPI: Exocrine Pancreatic Insufficiency*
