# Supplementary material for: Assessment of Weight Loss and Gastrointestinal Symptoms Suggestive of Exocrine Pancreatic Dysfunction After Acute Pancreatitis
Source: Clin Transl Gastroenterol. 2020 Dec 15;11(12):e00283. doi: 10.14309/ctg.0000000000000283 (PMC7743841; doi:10.14309/ctg.0000000000000283)
Supplement: SUPPLEMENTARY MATERIAL [file ct9-11-e00283-s002.docx]

**Supplementary Table 2.** Univariate analysis of factors associated with **weight loss at 3 months** in patients with follow-up at both 3 and 12 months

| Variable | Level | Weight Loss <10% (n=102) | Weight Loss ≥10% (n=38) | Total (n=140) | p-value |
| --- | --- | --- | --- | --- | --- |
| Age | Median (IQR) | 56 (40.2, 68.0) | 60 (47.5, 69.8) | 57.5 (43.8, 69.2) | 0.24 |
| BMI | Median (IQR) | 28 (24.3, 31.8) | 29.6 (25.3, 35.7) | 28.5 (24.7, 33.5) | 0.14 |
| Sex | Male | 42 (41.2) | 17 (44.7) | 59 (42.1) | 0.85 |
| Race | Caucasian | 94 (92.2) | 33 (89.2) | 127 (91.4) |  |
|  | AA | 8 (7.8) | 4 (10.8) | 12 (8.6) | 0.83 |
|  | Unknown | 0 | 1 | 1 |  |
| Etiology | Billiary | 50 (49.0) | 19 (50.0) | 69 (49.3) |  |
|  | Post-ERCP | 13 (12.7) | 3 (7.9) | 16 (11.4) |  |
|  | Other | 11 (10.8) | 3 (7.9) | 14 (10.0) |  |
|  | HTG | 9 (8.8) | 1 (2.6) | 10 (7.1) |  |
|  | Idiopathic | 11 (10.8) | 9 (23.7) | 20 (14.3) |  |
|  | Alcoholic | 8 (7.8) | 3 (7.9) | 11 (7.9) | 0.36 |
| Active Alcohol | No | 63 (61.8) | 24 (63.2) | 87 (62.1) |  |
|  | Yes | 39 (38.2) | 14 (36.8) | 53 (37.9) | 1.0 |
| Active Smoking | No | 79 (77.5) | 31 (81.6) | 110 (78.6) |  |
|  | Yes | 23 (22.5) | 7 (18.4) | 30 (21.4) | 0.77 |
| RAC | Mild | 74 (72.5) | 17 (44.7) | 91 (65.0) |  |
|  | Moderate | 22 (21.6) | 10 (26.3) | 32 (22.9) |  |
|  | Severe | 6 (5.9) | 11 (28.9) | 17 (12.1) | **<0.01** |
| AP | First AP | 71 (69.6) | 24 (63.2) | 95 (67.9) |  |
|  | RAP | 31 (30.4) | 14 (36.8) | 45 (32.1) | 0.64 |
| Total LOS | Median (IQR) | 6.0 (4.0, 9.0) | 11.5 (6.0, 18.0) | 7.0 (4.0, 12.0) | **<0.01** |
| Prior Diagnosis DM | No | 85 (83.3) | 30 (78.9) | 115 (82.1) |  |
|  | Yes | 17 (16.7) | 8 (21.1) | 25 (17.9) | 0.72 |
| New Diagnosis DM | No | 99 (97.1) | 32 (84.2) | 131 (93.6) |  |
|  | Yes | 3 (2.9) | 6 (15.8) | 9 (6.4) | **0.02** |
| New Diagnosis EPI | No | 100 (98.0) | 29 (78.4) | 129 (92.8) |  |
|  | Yes | 2 (2.0) | 8 (21.6) | 10 (7.2) | **<0.01** |
|  | missing | 0 | 1 | 1 |  |
| Taking Panc Enzymes | No | 101(99.0) | 29 (76.3) | 130 (92.9) |  |
|  | Yes | 1 (1.0) | 9 (24.3) | 10 (7.1) | **<0.01** |
| GI Symptoms of EPI | No | 16 (66.7) | 6 (75.0) | 22 (68.8) |  |
|  | Yes | 8 (33.3) | 2 (25.0) | 10 (31.2) | 1.0 |

* *n=32 who completed questionnaire at 3 months from cohort of 140. BMI: Body Mass Index; AP: Acute Pancreatitis;RAP: Recurrent Acute Pancreatitis; RAC: Revised Atlanta Criteria; LOS: Length of Stay; DM: Diabetes Mellitus; EPI: Exocrine Pancreatic Insufficiency*

*p-values are for univariate analysis comparing the groups with and without ≥10% total body weight loss at 3 months after AP attack.*
