# Supplementary material for: Assessment of Weight Loss and Gastrointestinal Symptoms Suggestive of Exocrine Pancreatic Dysfunction After Acute Pancreatitis
Source: Clin Transl Gastroenterol. 2020 Dec 15;11(12):e00283. doi: 10.14309/ctg.0000000000000283 (PMC7743841; doi:10.14309/ctg.0000000000000283)
Supplement: SUPPLEMENTARY MATERIAL [file ct9-11-e00283-s003.docx]

**Supplementary Table 3.** Multivariable Analysis of independent predictors of weight loss of ≥ 10% of total body weight at 3 months compared to baseline

|  | Adjusted OR | 95% CI | P-Value |
| --- | --- | --- | --- |
| Female Sex | 0.62 | 0.33-1.14 | 0.127 |
| BMI | 1.04 | 1.10-1.08 | **0.023** |
| Total Length of Stay | 1.05 | 1.02-1.09 | **0.001** |
| Prior DM | 2.16 | 1.03-4.57 | **0.042** |

BMI: Body Mass Index; DM: Diabetes Mellitus. All variables included are those that remained in the model (p<0.20) through backwards stepwise logistic regression.
